# A rare case of for synchronous advanced cancer of ascending colon and urinary bladder with simultaneous laparoscopic resection: A case report

**DOI:** 10.1016/j.ijscr.2018.11.056

**Published:** 2018-11-24

**Authors:** Toru Imagami, Satoru Takayama, Satoshi Kurokawa, Taku Hattori, Ryohei Matsui, Masaki Sakamoto, Hisanori Kani, Tsuyoshi Fujiwara

**Affiliations:** a Department of Surgery, Nagoya Tokushukai General Hospital, Kasugai City, Japan; b Department of Urology, Nagoya Tokushukai General Hospital, Kasugai City, Japan

**Keywords:** CT, computed tomography, Multiple primary malignant tumors, Synchronous colorectal and bladder cancer, Simultaneous laparoscopic resection

## Abstract

**Introduction:**

Advances in diagnostic techniques have resulted in an increase in the diagnosis of numerous patients with multiple primary cancers. However, the diagnosis of synchronous primary colorectal cancer and bladder cancer remains rare.

**Presentation of case:**

A 69-year-old man diagnosed with synchronous advanced cancer of the ascending colon and urinary bladder underwent simultaneous laparoscopic resection. His postoperative course was similar to that of routine colorectal cancer. The patient refused adjuvant therapy. The patient was diagnosed as having recurrence of bladder cancer 3 months after surgery; he died 9 months after surgery.

**Discussion:**

For multiple primary malignant tumors, simultaneous tumor resection is preferred. Simultaneous laparoscopic resection may be proposed for postoperative multidisciplinary treatment. If an established regimen is determined in the future, neoadjuvant chemotherapy may be an option for the treatment of synchronous advanced cancer.

**Conclusion:**

Simultaneous laparoscopic surgery is a greatly beneficial approach for synchronous cancer requiring multidisciplinary treatment. Additionally, an appropriate support system for patients is indispensable for completing multidisciplinary treatment.

## Highlights

• The case of synchronous primary colorectal cancer and bladder cancer remains rare.
• Multidisciplinary treatment is required for synchronous advanced colorectal cancer and bladder cancer.
• Simultaneous laparoscopic resection may be proposed for postoperative multidisciplinary treatment.

## 1 Introduction

Laparoscopic colectomy was first reported in 1991 [1], and laparoscopic surgery for colorectal cancer has become widespread in recent years. Laparoscopic radical cystectomy is a safe, feasible, and minimally invasive alternative to open radical cystectomy that has fewer overall complications, more reliable pathological and oncological efficacy, and shorter recovery time [2,3]. Advances in diagnostic techniques have resulted in an increase in the diagnosis of numerous patients with multiple primary cancers. However, literature describing the clinical course of synchronous primary colorectal cancer and bladder cancer are limited [[4], [5], [6]]. We present a case involving simultaneous laparoscopic surgery for synchronous advanced cancer of the ascending colon and urinary bladder. We describe the clinical course and review selected literature. This report conforms to the SCARE criteria [7].

## 2 Presentation of case

**Fig. 1:**
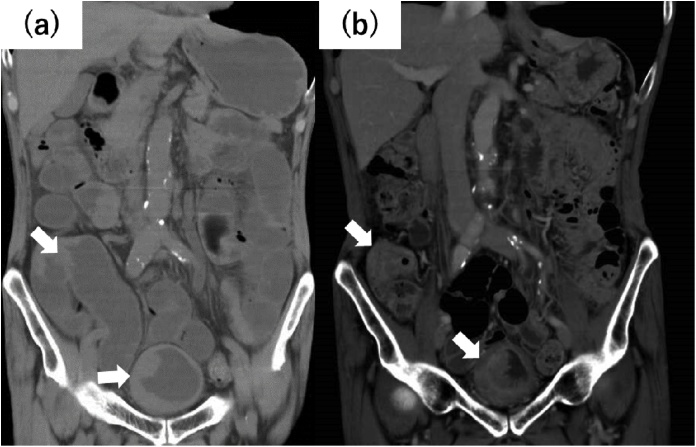
(a) Abdominal CT revealed construction findings of ileocecal and irregularity of the urinary bladder wall (white arrow). (b) Contrast-enhanced CT revealed a strong contrast effect (white arrow).

A 69-year-old man was referred to the emergency department because of vomiting, abdominal pain, and hematuria. His medical, surgical, and familial histories were unremarkable. Abdominal computed tomography (CT) revealed ileocecal bowel obstruction and irregularity in the urinary bladder wall (Fig. 1a). On hospitalization, a surgeon inserted an ileus tube for decompression. Colonoscopy confirmed a constricting tumor of the ascending colon that was pathologically diagnosed as adenocarcinoma. Cystoscopy revealed an irregular mucosa, but bladder dilation was not obtained despite saline injection. The urologist was unable to observe in detail and failed to perform a biopsy. Instead, bladder washing cytology was performed, which led to a diagnosis of urothelial carcinoma.

The ileus tube was removed 5 days after insertion. However, he experienced difficulty in eating because of abdominal pain and nausea. His hematuria persisted after hospitalization and required transfusion. For diagnosis of stage, contrast-enhanced CT was performed (Fig. 1b), which suggested lymph node metastasis (No #201, #283); however no distant metastasis was observed. The preoperative diagnosis was adenocarcinoma of the ascending colon (sT3N1M0) and urothelial carcinoma of the bladder (sT3N1M0).

The surgeons and urologists consulted and noted the following aspects: 1) For a patient receiving fasting therapy and transfusion, neoadjuvant chemotherapy is considered difficult. 2) For oral intake, resection of ascending colon cancer or bypass surgery is necessary; the former is a superior treatment for ascending colon cancer, and the perioperative course appears to remain unchanged in either surgery. 3) Urinary diversion is necessary to prevent the progression of anemia due to hematuria, and ileal conduit urinary diversion maintains the patient’s postoperative quality of life. 4) Laparoscopic surgery is advantageous for achieving early postoperative recovery. 5) When performing laparoscopic resection of ascending colon cancer and ileal conduit urinary diversion, the perioperative course tends to remains mostly unchanged despite the addition of laparoscopic cystectomy.

**Fig. 2:**
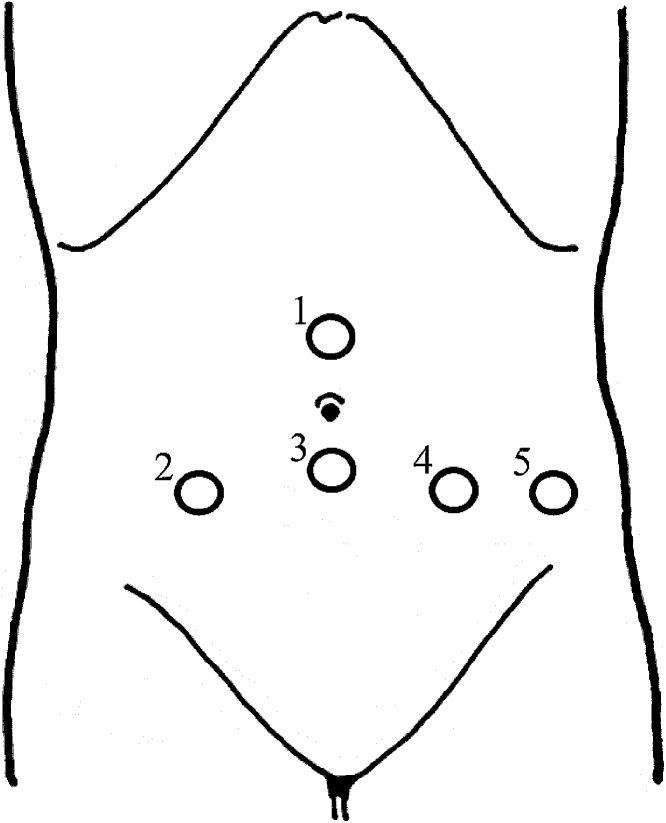
Circles show port arrangement. No 2 and 3 are 12 mm port and No 1, 4, 5 are 5 mm port. No 1 is 2.5 cm cranial side from umbilicus. No 3 is 2.5 cm caudal side from umbilicus. An ileal urinal conduct was constructed at No 2 post site.

These considerations led to the decision to perform simultaneous laparoscopic right hemicolectomy and radical cystectomy. With the patient in the lithotomy position under general anesthesia, ports were placed as shown in Fig. 2. The surgeons performed D3 lymph node dissection and mobilization, following which the urologists performed a radical cystectomy and pelvic lymph node dissection. A mini-laparotomy was performed by incisional extension from port sites 1–3, creating an incision of 7 cm. The surgeons and urologists worked in unison to perform a right hemicolectomy and ileal conduit urinary diversion. The duration of surgery was 556 min with a blood loss of 50 mL. Lymph node metastasis was pathologically observed in the left obturator external iliac lymph nodes.

The pathological diagnosis was stage T3N0M0 adenocarcinoma of the ascending colon and stage T4aN1M0 urothelial carcinoma of the urinary bladder. The only postoperative complication was wound infection of Clavien–Dindo Classification I. The infusion was terminated 7 days after surgery, which was the same postoperative course as the standard colorectal cancer resection. The urologist recommended adjuvant therapy, but the patient refused because he was not accustomed to the lifestyle changes required by having undergone a urostomy.

**Fig. 3:**
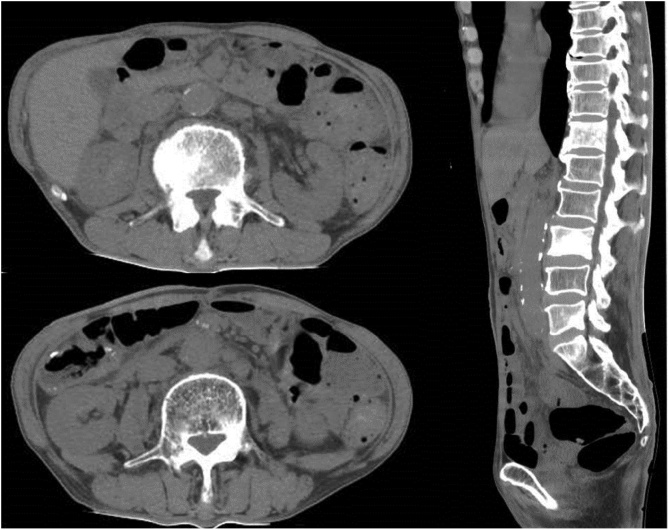
Three months after surgery, abdominal CT revealed metastatic tumor in para-aortic lymph nodes and spine (12th thoracic spine, 3th lumber spine and 5th lumber spine).

Follow-up CT conducted 3 months after the patient’s refusal to undergo adjuvant therapy revealed a metastatic tumor in the spine and para-aortic lymph nodes (Fig. 3), and the patient was diagnosed as having a recurrence of bladder cancer. The patient did not want to undergo chemotherapy with cisplatin and gemcitabine because of concerns regarding side effect. However, following a consultation with the urologist, the patient agreed to chemotherapy with gemcitabine alone. The overall response was diagnosed as progressive disease. The patient died 9 months after surgery.

## 3 Discussion

It is well recognized that cancers develop because of accumulated alterations of multiple responsible genes. Therefore, cancer-bearing patients are assumed to be at an increased risk of developing cancers in other organs [8]. The large intestine is the most common site for the development of multiple primary malignant tumors [8,9]. However, cases of synchronous advanced cancer of colorectal and bladder are rare, and there is no consensus on treatment.

The present case involved synchronous ascending colon cancer with obstruction and bladder cancer with lymph node metastasis. For stage IV bladder cancer, systemic chemotherapy is common. However, in the present case, progression of anemia due to hematuria was observed, and urinary diversion was considered necessary for bleeding control. In addition, oral intake was impossible because of colon obstruction and the possibility of resuming oral intake by systemic chemotherapy was miniscule. We have previously reported good surgical results of simultaneous endoscopic surgery for synchronous colorectal and genitourinary cancer [10]. On the basis of those findings, we decided to perform simultaneous resection aiming at recovery of oral intake, control of hematuria, and to plan adjuvant therapy.

Wadhwa had reported that combined procedures in laparoscopic surgery are feasible and appear to have several advantages in simultaneous management of two different coexisting pathologies without significant addition in postoperative morbidity and hospital stay [11]. To the best our knowledge, this has not been proven for simultaneous laparoscopic surgery conducted for synchronous colorectal cancer and bladder cancer. We simultaneously performed a laparoscopic right-hemi colectomy, a total cystectomy, and an ileal conduit urinary diversion. The postoperative course was similar to that of colorectal cancer surgery alone as reported by Wadhwa. From the patient’s general condition, it was considered that adjuvant therapy could be started 2 weeks postoperatively. Early recovery from such major surgery is an advantage of laparoscopic surgery and we consider that simultaneous laparoscopic resection is suitable for advanced synchronous colorectal and bladder cancer.

In the present case, we considered that ileal conduit urinary diversion is the best method to maintain the patient’s quality of life. The Japanese guidelines state that the acceptance of urinary diversion is not necessarily beneficial for elderly individuals. In the present case as well, contrary to our expectation, the patient could not accept the ileal conduit urinary diversion, which led to his refusal of adjuvant therapy. In limited cases, there have been reports that good surgical results were obtained in combination with neoadjuvant and/or adjuvant systemic chemotherapy of bladder cancer with metastasis [[12], [13], [14]]. Additionally, the effect of adjuvant radiotherapy for advanced bladder cancer was expected to be beneficial in several reports [[14], [15], [16]]. We regret that we were unable to complete this treatment, which was expected to be efficacious. An adequate support system for patients was considered necessary for multidisciplinary treatment.

If there were effective drugs for both cancers, we could have selected neoadjuvant chemotherapy. However, chemotherapy for synchronous cancer has not yet been established. Therefore, we believe that performing simultaneous surgery and planning to provide adjuvant therapy were appropriate choices. Given the rarity of these cases, accumulation of more cases and reports in the literature is needed to establish the best treatment for multiple primary cancers.

## 4 Conclusion

We believe that simultaneous laparoscopic surgery has a great advantage for synchronous cancer requiring multidisciplinary treatment. In addition, an appropriate support system for patients is indispensable for completing multidisciplinary treatment. Further reports of similar cases are required to establish the best treatment strategy for synchronous primary advanced cancer.

## Conflicts of interest

The authors have no conflict of interest to declare.

## Sources of funding

This research did not receive any specific grant from funding agencies in the pubic, commercial, or not-for-profit sectors.

## Ethical approval

This report was approved by the ethics committee in Nagoya Tokushukai General Hospital (Institutional Review Board approval 2018-08-003).

## Consent

Consent was obtained from patient’s guardian for publication of this case report.

## Author contributions

TI, ST and SK performed operation. TI drafted the manuscript. ST and SK participated in the correction of the manuscript. All authors read and approved the final manuscript.

## Registration of research studies

No research study involved in this case report. Not applicable.

## Guarantor

Toru Imagami.

## Provenance and peer review

Not commissioned, externally peer reviewed.
